# The impact of pulmonary function tests on early postoperative complications in open lung resection surgery: an observational cohort study

**DOI:** 10.1038/s41598-022-05279-8

**Published:** 2022-01-24

**Authors:** Ji Won Choi, Heejoon Jeong, Hyun Joo Ahn, Mikyung Yang, Jie Ae Kim, Duk Kyung Kim, Sang Hyun Lee, Keoungah Kim, Jisun Choi

**Affiliations:** 1 Department of Anesthesiology and Pain Medicine, Samsung Medical Center, Sungkyunkwan University School of Medicine, 81 Irwon-ro, Gangnam-gu, Seoul, 06351 South Korea; 2 Department of Anesthesiology, School of Dentistry, Dankook University, Cheonan, South Korea

**Keywords:** Medical research, Risk factors

## Abstract

We investigated whether pulmonary function tests (PFTs) can predict pulmonary complications and if they are, to find new cutoff values in current open lung resection surgery. In this observational study, patients underwent open lung resection surgery at a tertiary hospital were analyzed (n = 1544). Various PFTs were tested by area under the receiver-operating characteristic curve (AUC_ROC_) to predict pulmonary complications until 30 days postoperatively. In results, PFTs were generally not effective to predict pulmonary complications (AUC_ROC_: 0.58–0.66). Therefore, we could not determine new cutoff values, and used previously reported cutoffs for post-hoc analysis [predicted postoperative forced expiratory volume in one second (ppoFEV_1_) < 40%, predicted postoperative diffusing capacity for carbon monoxide (ppoDL_CO_) < 40%]. In multivariable analysis, old age, male sex, current smoker, intraoperative transfusion and use of inotropes were independent risk factors for pulmonary complications (model 1: AUC_ROC_ 0.737). Addition of ppoFEV_1_ or ppoDL_CO_ < 40% to model 1 did not significantly increase predictive capability (model 2: AUC_ROC_ 0.751, *P* = 0.065). In propensity score-matched subgroups, patients with ppoFEV_1_ or ppoDL_CO_ < 40% showed higher rates of pulmonary complications [13% (21/160) vs. 24% (38/160), *P* = 0.014], but no difference in in-hospital mortality [3% (8/241) vs. 6% (14/241), *P* = 0.210] or mean survival duration [61 (95% CI 57–66) vs. 65 (95% CI 60–70) months, *P* = 0.830] compared to patients with both > 40%. In conclusion, PFTs themselves were not effective predictors of pulmonary complications. Decision to proceed with surgical resection of lung cancer should be made on an individual basis considering other risk factors and the patient's goals.

## Introduction

Accurate assessment of lung function has been regarded as vital for prevention of pulmonary complications after lung resection^1^. Predicted postoperative forced expiratory volume in 1 s (ppoFEV_1_) and predicted postoperative diffusing capacity for carbon monoxide (ppoDL_CO_) are commonly used to predict pulmonary complications in thoracic surgery^1^. Many previous studies from the 1980s through the 2000s observed that morbidity and mortality significantly increased in patients whose ppoFEV_1_ or ppoDL_CO_ were < 40% of predicted normal values^2–5^, and patients with limited pulmonary function are often denied curative resection due to the possibility of postoperative respiratory failure.

However, the 40% cutoff points were not determined by objective analysis and were generally empirical and based on expert opinion^2–5^. Currently, many patients with ppoFEV_1_ or ppoDL_CO_ < 40% undergo surgery with acceptable morbidity and mortality, especially when minimally invasive techniques are used^6^. Notably, one study suggested that FEV_1_ and DL_CO_ are not associated with pulmonary complications in patients who had lobectomy using video-assisted thoracoscopic surgery (VATS)^7^. Accordingly, lower cutoff such as 30% ppoFEV_1_ and ppoDL_CO_ is being increasingly used in many institutions^8^. However, the previous cutoff points for ppoFEV_1_ and ppoDL_CO_ remain widely accepted for open lung resection surgery. With modern advances in anesthetic, surgical, and postoperative care, the predictive capability of pulmonary function tests (PFTs) needs to be re-evaluated for open lung resection surgery, especially when we consider that two-year survival was higher (62% vs. 18%) in patients who had surgery compared to those who were denied an operation in this high-risk group^9^.

Therefore, we performed an observational cohort study in patients who underwent open lung resection surgery for non-small cell lung cancer. Our primary objectives were to determine whether various PFTs can predict pulmonary complications within 30 postoperative days, and if they are, to find new cutoff values in open lung resection surgery. Whether PFTs are related to in-hospital mortality and long-term survival were also analyzed.

## Methods

### Study design

This was a single-center, observational cohort study to find the new cutoff values of PFTs which are related to pulmonary complications for open lung resection surgery. The Samsung Medical Center Institutional Review Board approved this study and waived the requirement for informed patient consent (Approval No. SMC 2018-12-056-001). The study proposal and statistical plan were registered at the Institutional Review Board before accessing patient data. The study was conducted in accordance to the original protocol. We adhered to the Strengthening the Reporting of Observational Studies in Epidemiology checklist for reporting observational studies. All methods were performed in accordance with the ethical principles of the 1964 Declaration of Helsinki and its later amendments.

### Patient records

We collected study data from electronic medical records at our institution from patients who had an open lobectomy or more extensive open thoracotomy for non-small cell lung cancer between January 2009 and December 2013 (n = 1544). Patients were followed up for mortality until December 31, 2015. Metastasectomy cases and those featuring concomitant surgery with other departments were excluded. The primary objective was to identify pulmonary complications occurring until 30 days postoperatively.

The following information was collected from electronic medical records: preoperative data included PFTs, age, sex, body mass index (BMI), comorbidities, American Society of Anesthesiologists (ASA) physical status, smoking, alcohol consumption, previous lung diseases, pulmonary tuberculosis, neoadjuvant therapy, cancer cell types and clinical tumor node metastasis (TNM) stages, and plan for surgery and postoperative analgesia. Comorbidities included hypertension, diabetes mellitus, renal dysfunction (estimated glomerular filtration rate < 60 ml/min/1.73 m^2^), cerebrovascular disease, cardiac disease, and pulmonary disease. Cerebrovascular disease included cerebral infarction, cerebral hemorrhage, dementia, Parkinson’s disease, and Alzheimer’s disease. Cardiac disease included coronary artery disease and heart failure. Underlying pulmonary disease included chronic obstructive pulmonary disease, bronchiectasis, asthma, and interstitial lung disease. Current smokers were defined as patients who were presently smoking or had stopped smoking within 1 month prior to surgery. Heavy drinking was defined as the consumption of ≥ 15 drinks per week for men and ≥ 8 drinks per week for women, in accordance with Centers for Disease Control and Prevention guidelines (https://www.cdc.gov/alcohol/fact-sheets/alcohol-use.htm).

Intraoperative data collected included the duration of surgery, requirement for transfusion, use of hydroxyethyl starch, and use of continuous inotropes (dopamine, dobutamine, or epinephrine continuous infusion) or vasopressors (phenylephrine or norepinephrine continuous infusion) during operation.

Postoperative data were collected from the institutional thoracic surgery registry and included the incidence rates of pulmonary and other complications, duration of intensive care unit (ICU) and hospital stays, and occurrence of in-hospital mortality and long-term survival. Other complications within the first 30 days included new-onset arrhythmia, myocardial infarction, renal complication, cerebral infarction, seizure, pulmonary thromboembolism, and surgical complications. Renal complication was defined as an Acute Kidney Injury Network classification ≥ 2. Surgical complications included prolonged air leak (≥ 5 days), prolonged effusion (≥ 5 days), chylothorax, vocal cord palsy, empyema, wound infection, wound dehiscence, and bronchopleural fistula.

### Pulmonary function tests

FEV_1_, DL_CO_, forced vital capacity (FVC), FEV_1_/FVC, and reduction in forced expiratory flow at 25–75% of the pulmonary volume (FEF_25-75_) were analyzed in this study. The values of FEV_1_ and DL_CO_ were expressed as a percentage of the values predicted by age, sex, and height^10^. Calculations of ppoFEV_1_ and ppoDL_CO_ were performed by multiplying FEV_1_ and DL_CO_ values by the percentage of functional lung tissue remaining after resection^11^. ppoFEV_1_ and ppoDL_CO_ were calculated based on anatomical resection. Blood gas analysis, six-minute walk tests, tests of expired gas analysis during exercise, and lung quantitative perfusion scans were not routinely performed; thus, these parameters were not analyzed in the current study.

### Primary endpoint: pulmonary complications

Pulmonary complications included pneumonia, acute respiratory distress syndrome (ARDS), and atelectasis requiring bronchoscopy within 30 days of surgery. ARDS was defined in accordance with the 2012 Berlin criteria, as acute (within 1 week of a known clinical insult) hypoxemic respiratory failure [(ratio of the partial pressure of arterial oxygen to the fraction of inspired oxygen (< 300 mm Hg)], with bilateral opacities on chest imaging not fully explained by cardiac failure or fluid overload^12^. Pneumonia was defined as meeting three of five characteristics: fever, leukocytosis, chest x-ray with infiltrate, positive culture from sputum, or treatment with antibiotics. Atelectasis was diagnosed only when bronchoscopy toileting was performed.

### Institutional protocol for anesthesia, surgery, and postoperative management

Most patients received balanced anesthesia, which was a combination of volatile anesthetic agents, non-depolarizing neuromuscular blocking agents, and continuous intravenous infusion of remifentanil. Lactated Ringer’s solution was used as maintenance fluid and was infused at a rate of 3–5 ml/kg/h. If intraoperative bleeding occurred, 5% human albumin (Green Cross, Gyeonggi, Korea) or 6% hydroxyethyl starch (Fresenius Kabi, Bad Homburg, Germany) was administered. Transfusion was performed for resuscitation if the transfusion cutoff was reached (hemoglobin < 8 g/dl). A protective ventilation protocol was implemented for all patients. Pulmonary resection was performed using standard posterolateral thoracotomy with mediastinal lymph node dissection. All patients were routinely extubated at the end of surgery unless the attending anesthesiologists or surgeons decided not to.

Patients remained in the ICU for one day for postoperative management. Analgesic methods were determined in accordance with each surgeon’s preference, as well as the existence of contraindications for regional analgesia. Maintenance fluid was administered at a rate of 1–2 ml/kg/h in the ICU and ward. Patients were encouraged to ambulate beginning on postoperative day one and also participated in a daily physiotherapy program, which included deep-breathing exercises, incentive spirometry, and chest physiotherapy delivered by physiotherapists and attending nurses during the ICU and ward stays.

### Statistical analysis

This observational cohort study did not conduct an a priori statistical power calculation and the sample size was based on the available data. Our primary objective was to determine whether various PFTs can predict pulmonary complications. We constructed receiver operating characteristic curves (ROC) using logistic regression model and assessed the predictive capacity of each PFT. Co-variates were not adjusted for primary ROC analysis. We defined tests showing an area under the ROC (AUC_ROC_) > 0.7 as optimal^13^. Pairwise comparisons of ROC curves for each PFT were conducted by DeLong’s method (hypothesis: true difference in AUC is not equal to 0)^14^.

For post hoc test, multivariable logistic regression was used to assess the risk factors for pulmonary complications. All preoperative and intraoperative variables were entered into analysis. The multivariable analysis was based on a backward stepwise method, with *P* < 0.05 for the inclusion of variables and *P* > 0.10 for the removal of variables. The goodness of fit was examined by the Hosmer–Lemeshow test. AUC_ROC_ was compared between models with and without PFT data.

Propensity score matching was also performed as a post hoc study. Groups were divided into below (ppoFEV_1_ < 40% or ppoDL_CO_ < 40%) or above (both ≥ 40%) according to the previous cutoff points for ppoFEV_1_ and ppoDL_CO_. Matched variables included age, sex, BMI, ASA physical status, current smoking, heavy drinking, underlying comorbidities, cell types, clinical TNM stage, neoadjuvant therapy, and plan for surgery and postoperative analgesia. One-to-one matching was performed using the nearest-neighbor method with a caliper width of 0.3 (pulmonary complications), 0.2 (in-hospital mortality), and 0.4 (long-term survival) in a pairwise manner. We analyzed pulmonary complications and in-hospital mortality between below and above groups using conditional logistic regression. Long-term survival between below and above groups was analyzed using the Kaplan–Meier method and stratified Cox regression. Stratum variable was a group (ppoFEV_1_ or ppoDL_CO_ < 40% vs. both > 40%). The proportional hazards assumption was tested by Schoenfeld residual graph. Participants who were not available due to follow-up loss or non-occurrence of outcome event were right censored for long-term survival analysis. Analyses of clinical end points were reported using available data with no imputation for missing data given the low observed rate of missingness (< 5%). An independent *t*-test or the Wilcoxon rank-sum test were used to determine significant differences in continuous variables. The chi-squared test or Fisher’s exact test was used to compare categorical variables. Patient demographic and clinical data are summarized as frequency (percentage) for categorical variables and mean ± standard deviation or median (interquartile range) for continuous variables. The normality of the data distribution was evaluated with the Shapiro–Wilk test. All reported *P* values were two-sided, and *P* < 0.05 was considered statistically significant. All statistical analyses were performed using MedCalc for Windows (version 7.3; MedCalc Software, Mariakerke, Belgium) or SAS (version 9.2; SAS Institute, Cary, NC, USA).

## Results

### Characteristics of patients, surgery, and complications

The analysis cohort comprised all 1544 patients who underwent an open lobectomy or more extensive open thoracotomy for lung cancer between January 2009 and December 2013 at a single institution. The surgeries included lobectomy (n = 1187), sleeve lobectomy (n = 180), and pneumonectomy (n = 177). Patients were followed for mortality until December 31, 2015. The median follow-up time was 40 months for long-term prognosis [range: 0 months (in-hospital mortality) to 83 months]. Baseline patient and operative characteristics compared between pulmonary complications (+) and (−) patients are shown in supplementary Table S1.

Outcome data including pulmonary and other complications are shown in supplementary Table S2. Pulmonary complications developed in 12% of patients (178/1544) [ARDS: 6% (96/1544), pneumonia: 6% (100/1544), and atelectasis requiring bronchoscopy: 2% (25/1544)]. The ICU and hospital stays were longer in pulmonary complications (+) patients than in (−) patients. In-hospital mortality rates were 0.7% (9/1366) and 17% (30/178) in pulmonary complications (−) and (+) patients, respectively.

### PFTs and pulmonary complications

**Figure 1:**
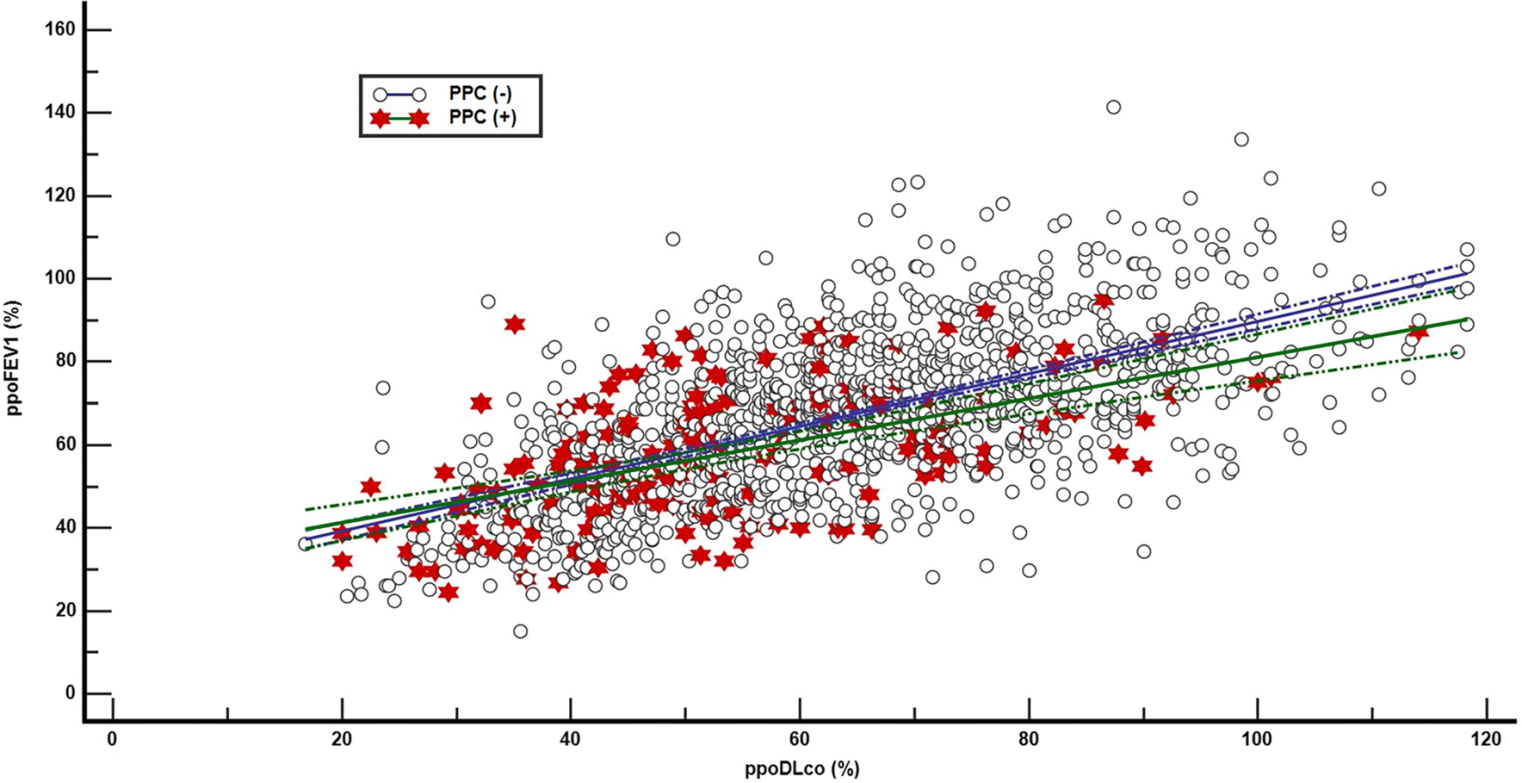
Scatter diagrams of ppoFEV_1_ and ppoDL_CO_ in the PPC (+) and (−) patients. Each dot indicates the ppoFEV_1_ and ppoDL_CO_ of one patient. Open circles indicate a PPC (−) patient. Filled stars indicate a PPC (+) patient. There is no difference between the scatter plots of PPC (+) and PPC (−) patients (*P* = 0.605). The diagonal line and accompanying dotted lines represent the regression line with 95 percent confidence limits, respectively. *ppoFEV*_*1*_ predicted postoperative expiratory volume in 1 s, *ppoDL*_*CO*_ predicted diffusing capacity for carbon monoxide, *PPCs* postoperative pulmonary complications.

Figure 1 shows scatter plots of ppoFEV_1_ and ppoDL_CO_ between pulmonary complications (+) and (−) patients; the distribution pattern of ppoFEV_1_ and ppoDL_CO_ did not differ between pulmonary complications (+) and (−) patients (*P* = 0.605). Patients with pulmonary complications were scattered widely rather than being concentrated in the left lower corner of the scatter plots (i.e., ppoFEV_1_ and ppoDL_CO_ < 40%).

**Figure 2:**
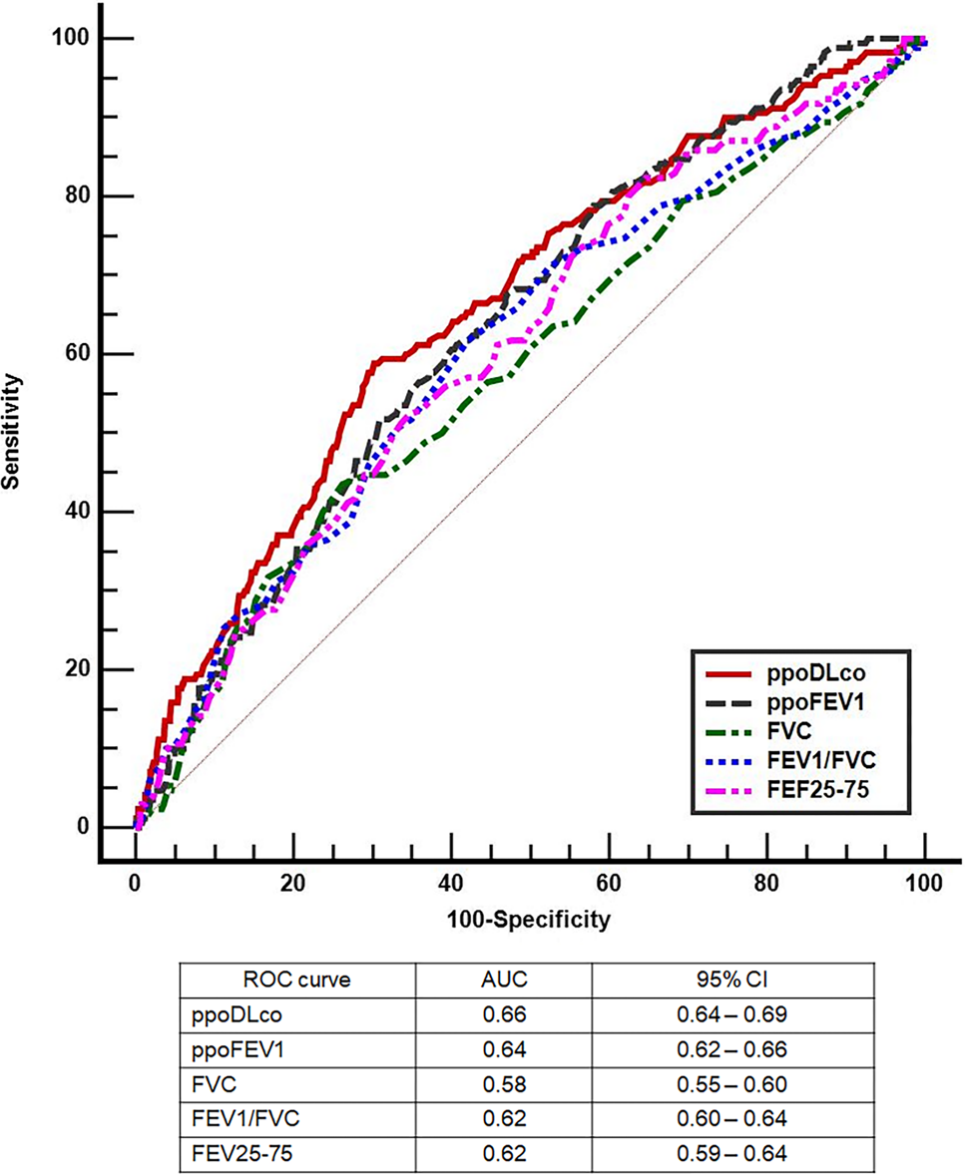
AUC_ROC_ of various pulmonary function tests. Pulmonary function tests performed poorly in predicting postoperative pulmonary complications (AUC_ROC_: 0.58–0.66). AUC_ROC_ values did not statistically differ from each other, except between ppoDL_CO_ and FVC (*P* = 0.010), and between ppoFEV_1_ and FVC (*P* = 0.005). *AUC*_*ROC*_ area under the receiver operating characteristic curve, *CI* confidence interval, *ppoFEV*_*1*_ postoperative expiratory volume in 1 s, *ppoDL*_*CO*_ diffusing capacity for carbon monoxide, *FEF25–75* a reduction in forced expiratory flow at 25–75% of the pulmonary volume, *FEV*_*1*_*/FVC* forced expiratory volume in 1 s/forced vital capacity.

The ROC curves of each PFT are shown in Fig. 2. PFTs performed poorly when predicting pulmonary complications (AUC_ROC_: 0.58–0.66). ppoDL_CO_ showed the highest AUC_ROC_ [0.66; 95% confidence interval (CI) 0.64, 0.69] among PFTs. The AUC_ROC_ of ppoFEV_1_ was 0.64 (95% CI 0.62, 0.66). For FVC, FEV_1_/FVC, and FEF_25–75_, the AUC_ROC_ was 0.58 (95% CI 0.55, 0.60), 0.62 (95% CI 0.60, 0.64), and 0.62 (95% CI 0.59, 0.64), respectively. AUC_ROC_ values did not statistically differ from each other, except between ppoDL_CO_ and FVC (*P* = 0.010), and between ppoFEV_1_ and FVC (*P* = 0.005). Combining ppoFEV_1_ and ppoDL_CO_ (AUC_ROC_ 0.67; 95% CI 0.63, 0.71) did not improve discrimination of pulmonary complications compared with ppoDL_CO_ alone (AUC_ROC_ 0.66; *P* = 0.297).

Because PFTs were not effective predictors of pulmonary complications, we could not present new cutoff points for preoperative PFTs. We used previous cutoffs for post-hoc analysis.

### Post-Hoc analysis: multivariable analysis for pulmonary complications

**Figure 3:**
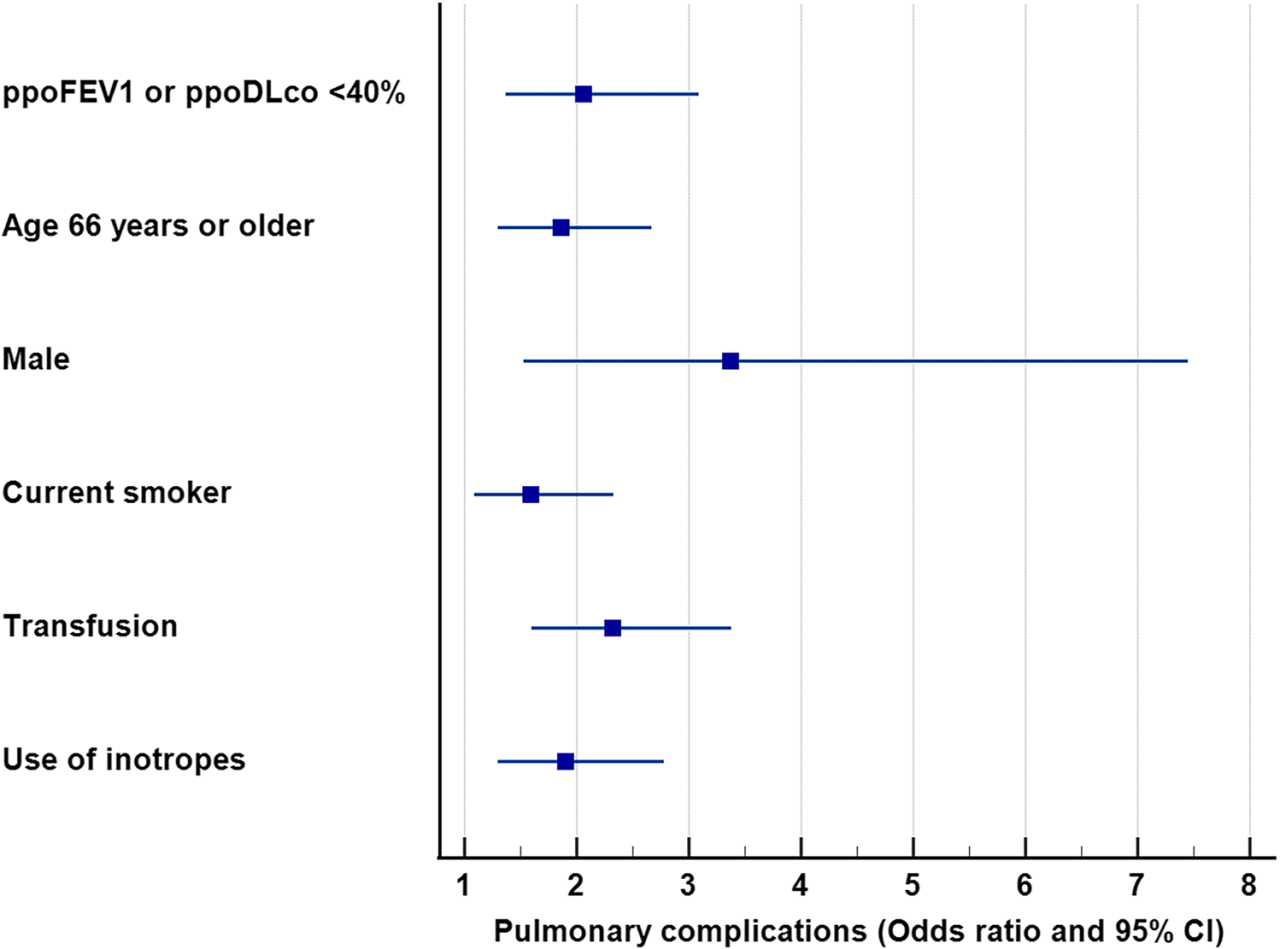
Independent association between risk factors and postoperative pulmonary complications. *ppoFEV*_*1*_ predicted postoperative expiratory volume in 1 s, *ppoDL*_*CO*_ diffusing capacity for carbon monoxide.

Multivariable logistic regression was performed to identify the risk factors for pulmonary complications using the previous cutoffs (dependent variable: presence of pulmonary complications, supplementary Table S3). Independent co-variates for pulmonary complications were age ≥ 66 years, male sex, current smoker, intraoperative transfusion and use of inotropes. The AUC_ROC_ was 0.737 (95% CI 0.695, 0.778) without PFT data (Model 1). ppoFEV_1_ or ppoDL_CO_ < 40% was an independent risk factor in multivariable analysis (Fig. 3) but tis adddition to other co-variates did not significantly increase the predictive capability [Model 2, AUC_ROC_ 0.751 (95% CI 0.712, 0.791); Model 1 vs. model 2, *P* = 0.065; supplementary Fig. S2, supplementary Table S3].

### Post-Hoc analysis: propensity score matching

**Figure 4:**
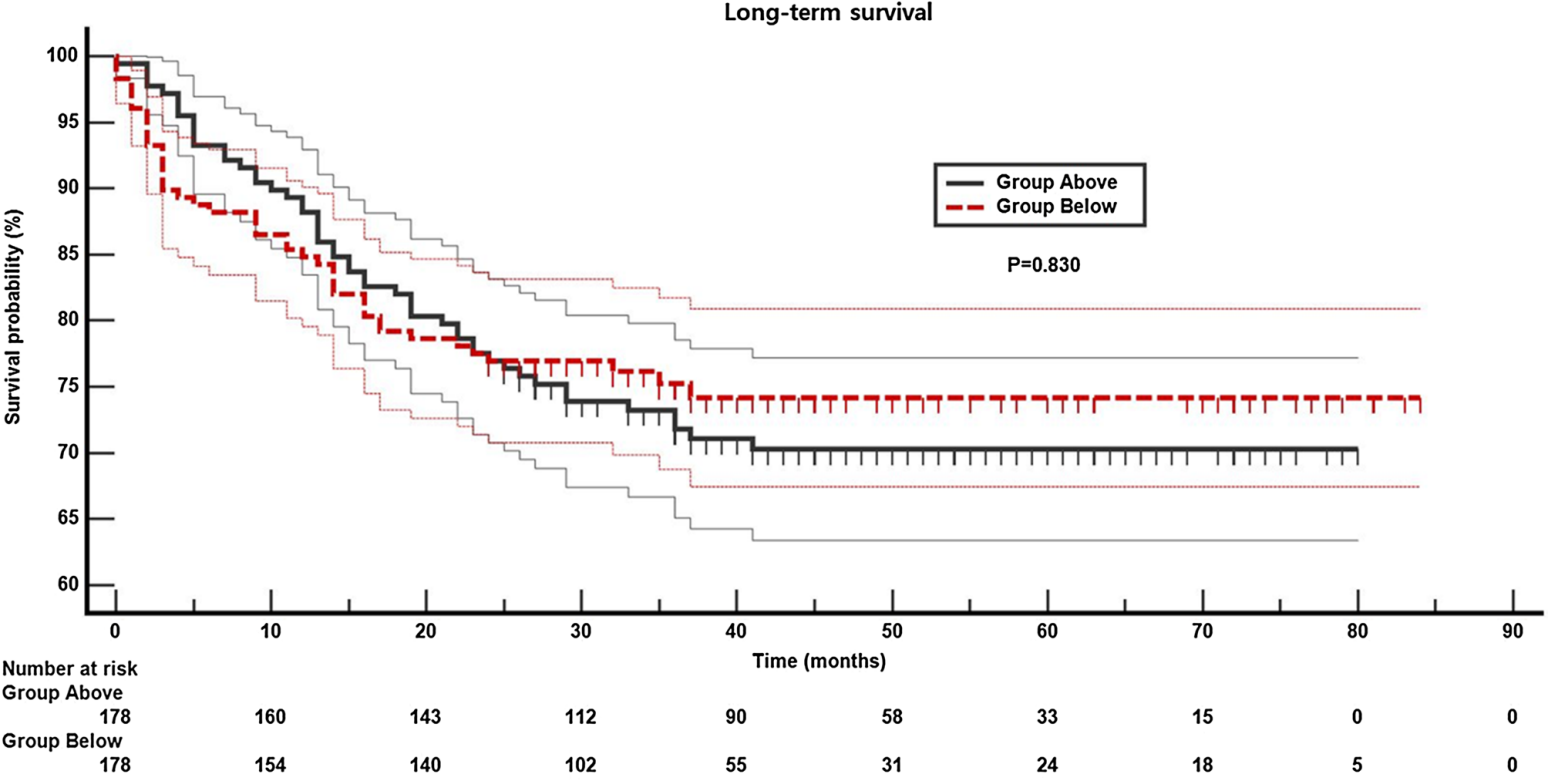
Kaplan–Meier survival analysis of the above (ppoDL_CO_ ≥ 40% and ppoFEV_1_ ≥ 40%) and below (ppoDL_CO_ < 40% or ppoFEV_1_ < 40%) groups in the propensity score-matched data. There was no difference between the two groups (*P* = 0.830, hazard ratio 0.95, 95% confidence interval: 0.62, 1.47). *ppoFEV*_*1*_ postoperative expiratory volume in 1 s, *ppoDL*_*CO*_ diffusing capacity for carbon monoxide.

Patients were divided into two groups, Above (ppoDL_CO_ ≥ 40% and ppoFEV_1_ ≥ 40%) and Below (ppoDL_CO_ < 40% or ppoFEV_1_ < 40%), and propensity score matching was performed to balance confounding factors between the two groups (Supplementary Table S4, Supplementary Fig. S1). After propensity score matching, pulmonary complications were higher in Below group compared to Above group [24% (38/160) vs. 13% (21/160); OR 2.4 (95% CI 1.2, 4.7); *P* = 0.014]. However, there was no difference in in-hospital mortality [Above vs. Below groups: 3% (8/241) vs. 6% (14/241); OR 1.75 (95% CI 0.74, 4.17); *P* = 0.210]. For long-term survival, the Kaplan–Meier survival analysis showed no difference between Above and Below groups; the mean survival duration was 61 (95% CI 57, 66) months and 65 (95% CI 60, 70) months (hazard ratio 0.95, 95% CI 0.62, 1.47; *P* = 0.830) in Above and Below groups, respectively (Fig. 4).

## Discussion

In the current study, PFTs themselves were not effective predictors of pulmonary complications in open lung resection surgery; therefore, we could not present new cutoff points for preoperative PFTs.

In this study, we used ROC analysis, which has not been employed previously in relation to PFTs. Based on the ROC curves (Fig. 2) and scatter plots of ppoFEV_1_ and ppoDL_CO_ (Fig. 1), we conclude that the performance of various PFTs for the prediction of pulmonary complications is unsatisfactory. Considering that we place great emphasis on ppoFEV_1_ and ppoDL_CO_ for surgical decision-making, this is quite disappointing; however, these results may explain why patients with low ppoFEV_1_ and ppoDL_CO_ often have good postoperative outcomes^6,9,15,16^.

The reason for poor predictability of PFTs seems to be the existence of other risk factors of equal or greater importance such as operative factors, and patient's other comorbidities. Age ≥ 66 years (OR 1.86), male sex (OR 3.37), current smoker (OR 1.59), all had a significant impact on pulmonary complications in multivariable analysis. Intraoperative events such as transfusion (OR 2.32) and use of inotropes (OR 1.90) also had a great influence on pulmonary complications (Fig. 3, supplementary Table S3). Another reason is lack of information on cardiopulmonary interaction in the current PFTs. To evaluate pulmonary function in relation to cardiac reserve, physiologic tests, such as the six-minute walk tests or expired gas analysis during exercise test are required, but these physiologic tests were not included in the present studies and are only selectively recommended for patents with poor PFTs in the current guidelines^3,4,9^. These physiologic pulmonary tests, however, are increasingly regarded as a more important predictor of morbidities in thoracic surgery patients^17,18^.

Previously, the influence of PFTs on patient outcomes has been controversial. Decades ago, several studies showed higher complication rates or mortality after lobectomy in patients with low ppoDL_CO_ or ppoFEV_1_^1,19–21^. One study even reported that the patients with ppoFEV_1_ or ppoDL_CO_ < 60% had significantly poorer short-and long-term postoperative outcomes in early-stage non-small cell lung cancer (retrospective, n = 391)^22^. However, in a more recent retrospective study, marginal PFT status was not associated with the development of a major complication following lobectomy and was not an independent predictor of mortality when other variables were controlled (n = 1259)^23^. All these previous reports included both VATS and open surgery.

When VATS and open thoracotomy were compared, FEV_1_ and DL_CO_ (< 60%) were predictors of pulmonary complications when lobectomy was performed through thoracotomy but not through VATS (n = 340)^7^, and VATS patients had a longer survival duration than those with open thoracotomy among patients with low ppoDL_CO_ or ppoFEV_1_ (< 40%) (n = 84, 54 vs. 20 months)^16^. However, these two retrospective studies were conducted between 1997 and 2009.

Our study is one of the largest observational studies (n = 1544) comprised of open lung resection surgery and includes a significant number of patients with ppoDL_CO_ or ppoFEV_1_ < 40% (n = 238). We observed that ppoFEV_1_ and ppoDL_CO_ themselves were not effective predictors of pulmonary complications, and addition of previous cutoff to other risk factors did not significantly increase the risk predictive capability of the model. In propensity score-matched subgroups, patients with ppoFEV_1_ or ppoDL_CO_ < 40% showed higher rates of pulmonary complications. However, we regard that the incidence (13% vs. 24%; *P* = 0.014) is acceptable considering poor PFTs in these patients. In addition, the higher incidence of pulmonary complications in these patients did not result in difference in in-hospital mortality. And in patients who survived the early postoperative period, the long-term survival was similar at the mean survival duration (61 months vs. 65 months, Above vs. Below groups, respectively). These findings support a surgical option in this high-risk group and is considered meaningful because two-year survival was higher (62% vs. 18%) in patients who had surgery compared to those who were denied an operation^9^.

An additional finding of the current study was that combining ppoFEV_1_ and ppoDL_CO_ did not result in superior prediction of pulmonary complications compared to ppoDL_CO_ alone. ppoFEV_1_ and ppoDL_CO_ are regarded as two separate measures of pulmonary function (ppoFEV_1_ for airway flow; ppoDL_CO_ for alveolar-vascular gas exchange); thus, the absence of an additive effect was unexpected. For possible explanations, some patients may not have performed the spirometric test properly; FEV_1_ is heavily dependent on patient cooperation^24^. In addition, in patients with moderate to severe chronic obstructive pulmonary disease, or with tumor-generated endobronchial obstruction, resection of the most affected parenchyma or relief of obstruction may actually improve FEV_1_ postoperatively^19,25–27^. In contrast, DL_CO_ is related to the total functioning surface area of the alveolar-capillary interface and is generally independent of patient effort. Therefore, it is a more sensitive measure of parenchymal damage than spirometric measurements^28^. This may be the reason that there was no additive effect between ppoFEV_1_ and ppoDL_CO_.

Our study has some limitations. First, this was a single-center study. Other hospitals with different patient care protocols may thus produce different results. Second, our study involved patients who had open lung resection surgery. Thus, more favorable results may be possible with less invasive thoracic surgeries, such as VATS. Third, we did not test 30% cutoff in our post-hoc analysis because only small number of patients belonged to this category (31 patients with a ppoFEV_1_ < 30%, 33 patients with a ppoDLco < 30%, and 12 patients with both values < 30%). Forth, there is a possibility of selection bias in our cohort. The surgeons were not blinded to the ppoFEV_1_ and ppoDL_CO_ values; thus, if surgery was offered to the patients whose ppoDL_CO_ and ppoFEV_1_ < 40%, it may be that those patients were relatively healthy with respect to comorbidities, or may have been offered less extensive surgery. We attempted to adjust for this possible bias by using propensity score matching.

In conclusion, PFTs themselves were not effective in predicting pulmonary complications, and the evidence supporting the decision to operate based on PFTs is limited. Previous cutoff 40% still has an influence in pulmonary complications after open lung resection surgery but no impact on mortality when other variables are controlled. Therefore, decision to proceed with surgical resection should be made on an individual basis with consideration of the patient's goals and physiologic state.

## Supplementary Information

Supplementary Information 1.

Supplementary Information 2.

Supplementary Information 3.

Supplementary Information 4.

Supplementary Information 5.

Supplementary Information 6.

Supplementary Information 7.

## Data Availability

The datasets generated during and analyzed during the current study are available from the corresponding author on reasonable request.
