# Supplementary figures and images for: The impact of pulmonary function tests on early postoperative complications in open lung resection surgery: an observational cohort study
Source: Sci Rep. 2022 Jan 24;12:1277. doi: 10.1038/s41598-022-05279-8 (PMC8786949; doi:10.1038/s41598-022-05279-8)

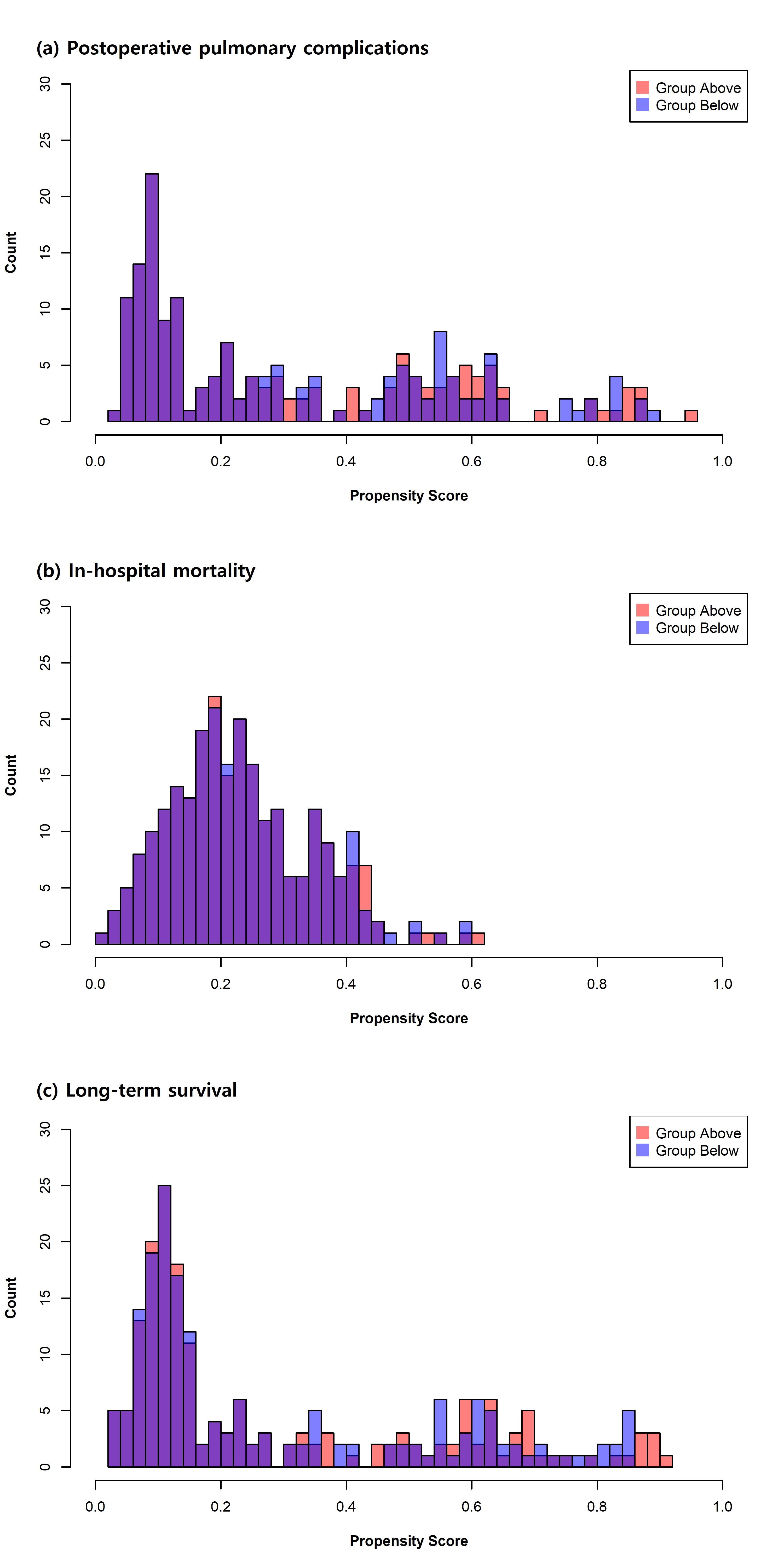

Supplement: Supplementary file 1 — Supplementary Information 1. [file 41598_2022_5279_MOESM1_ESM.tif]

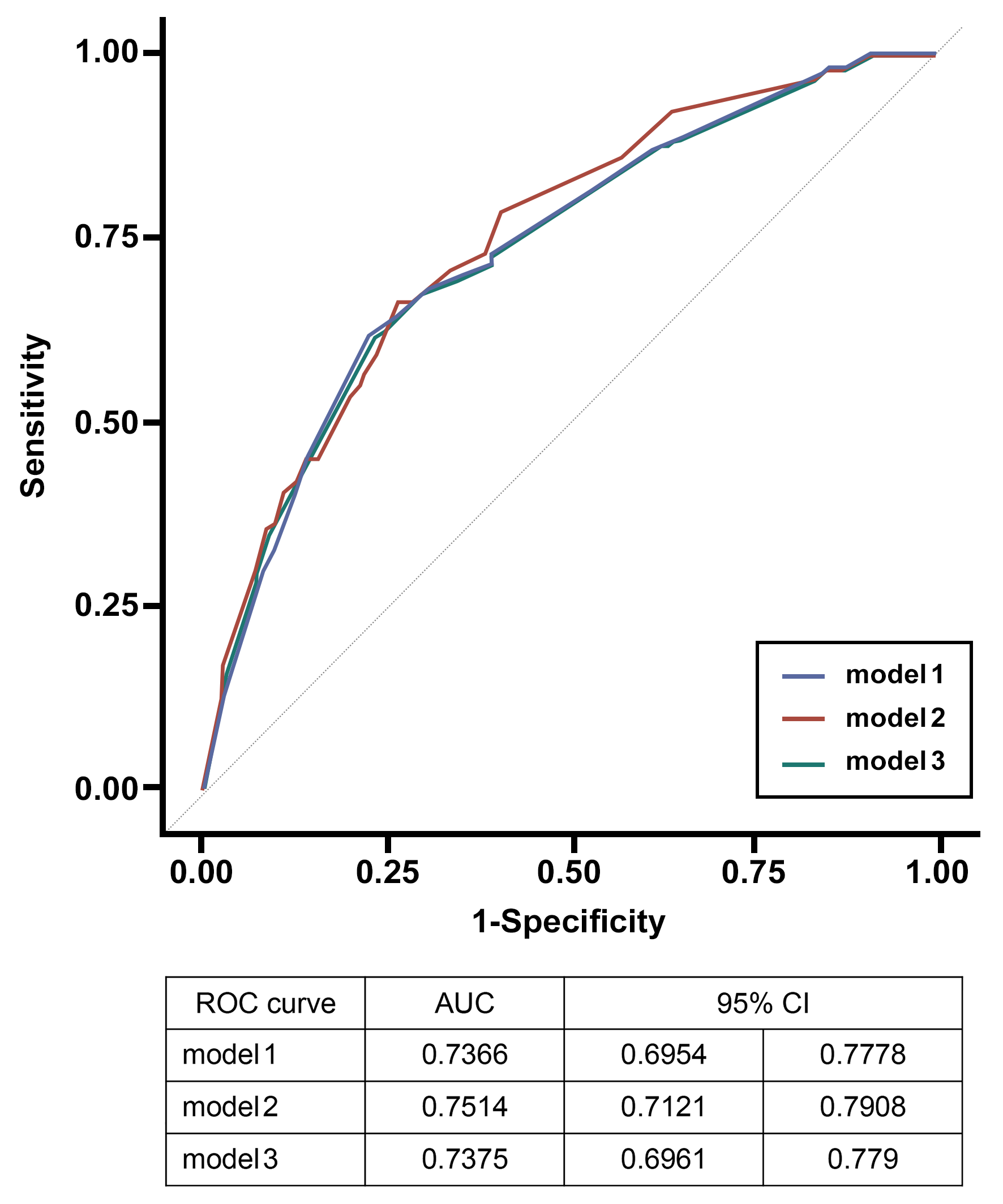

Supplement: Supplementary file 2 — Supplementary Information 2. [file 41598_2022_5279_MOESM2_ESM.tif]
