# Supplementary material for: The impact of pulmonary function tests on early postoperative complications in open lung resection surgery: an observational cohort study
Source: Sci Rep. 2022 Jan 24;12:1277. doi: 10.1038/s41598-022-05279-8 (PMC8786949; doi:10.1038/s41598-022-05279-8)
Supplement: Supplementary file 3 — Supplementary Information 3. [file 41598_2022_5279_MOESM3_ESM.docx]

| **Supplementary Table 1.** Baseline patient and operative characteristics, compared between the PPC (−) and PPC (+) groups. | | | | |
| --- | --- | --- | --- | --- |
| Variables | | PPCs (−)  (n = 1,366) | PPCs (+)  (n = 178) | *P* value |
| Preoperative data | |  |  |  |
| Age, yr | | 63 ± 9.5 | 67 ± 7.3 | < 0.001 |
| Sex, male | | 1102 (81) | 171 (96) | < 0.001 |
| Body mass index, kg/m^2^ | | 24 ± 3 | 23 ± 4 | 0.035 |
| ASA physical status ≥3 | | 117 (9) | 29 (16) | 0.001 |
| Current smoker | | 250 (23) | 53 (33) | 0.004 |
| Heavy drinking | | 156 (11) | 27 (15) | 0.183 |
| Cell types | |  |  | 0.005 |
| Squamous cell carcinoma | | 671 (49) | 110 (62) |  |
| Adenocarcinoma | | 581 (43) | 54 (30) |  |
| Etc. | | 114 (8) | 14 (8) |  |
| TNM Stage 3 & 4 | | 434 (32) | 61 (35) | 0.489 |
| Neoadjuvant CCRT | | 262 (19) | 43 (24) | 0.142 |
| Comorbid condition | |  | | |
| Hypertension | | 513 (37) | 66 (37) | 0.967 |
| Diabetes mellitus | | 219 (16) | 30 (17) | 0.863 |
| Previous lung operation | | 25 (2) | 6 (3) | 0.274 |
| Pulmonary lung disease | | 130 (10) | 35 (20) | < 0.001 |
| Cardiac disease | | 75 (6) | 8 (5) | 0.706 |
| Cerebrovascular disease | | 43 (3) | 12 (7) | 0.027 |
| Chronic renal disease | | 4 (0) | 0 (0) | > 0.999 |
| Pulmonary tuberculosis | | 171 (13) | 27 (15) | 0.381 |
| Pulmonary function tests, % predicted | | | | |
| ppoFEV_1_ | 67.8 ± 19.1 | | 58.7 ± 16.7 | < 0.001 |
| ppoDL_CO_ | 64.2 ± 18.7 | | 53.7 ± 17.8 | < 0.001 |
| FVC | 94.1 ± 16.1 | | 90.0 ±17.0 | 0.002 |
| FEV_1_ | 90.6 ± 19.8 | | 82.0 ± 18.3 | < 0.001 |
| FEV_1_/FVC | 69.6 ± 10.4 | | 65.1 ± 11.5 | < 0.001 |
| FEF25 – 75 | 64.6 ± 31.4 | | 52.4 ± 27.4 | < 0.001 |
| LVEF, % | 64.1 ± 6.0 | | 63.4 ± 6.6 | 0.148 |
| Intraoperative data |  | |  |  |
| Type of surgery |  | |  | 0.048 |
| Lobectomy | 1055 (77) | | 132 (74) |  |
| Sleeve lobectomy | 163 (12) | | 17 (10) |  |
| Left pneumonectomy | 102 (8) | | 16 (9) |  |
| Right pneumonectomy | 46 (3) | | 13 (7) |  |
| Transfusion | 326 (24) | | 96 (54) | < 0.001 |
| Use of hydroxyethyl starch | 553 (41) | | 109 (61) | < 0.001 |
| Use of inotrope | 388 (28) | | 100 (56) | < 0.001 |
| Use of vasopressor | 706 (49) | | 121 (64) | < 0.001 |
| Operation duration, min | 215.0 ± 72.8 | | 229.8 ± 72.0 | 0.021 |
| Operator (1/2/3/4/5/6/7), % | 32/27/12/14/13/2/0 | | 37/22/11/14/14/2/0 | 0.721 |
| Anesthesiologist (1/2/3/4), % | 32/29/20/19 | | 33/27/20/20 | 0.973 |
| Postoperative analgesia |  | |  | 0.119 |
| Thoracic epidural analgesia | 474 (35) | | 59 (33) |  |
| IV PCA | 462 (34) | | 50 (28) |  |
| Paravertebral block | 430 (31) | | 69 (39) |  |

Values are presented as mean ± standard deviation or n (%).

PPCs, postoperative pulmonary complications; ASA, American Society of Anesthesiologist; CCRT, concurrent chemoradiotherapy; TNM, tumor node metastasis; ppoDL_CO_, predictive postoperative diffusing capacity for carbon monoxide; ppoFEV_1_, predictive postoperative forced expiration volume in one second; FVC, forced vital capacity; FEF25-75, a reduction in forced expiratory flow at 25 – 75% of the pulmonary volume; LVEF, left ventricular ejection fraction; IV-PCA, intravenous patient controlled analgesia.
