# Supplementary material for: The impact of pulmonary function tests on early postoperative complications in open lung resection surgery: an observational cohort study
Source: Sci Rep. 2022 Jan 24;12:1277. doi: 10.1038/s41598-022-05279-8 (PMC8786949; doi:10.1038/s41598-022-05279-8)
Supplement: Supplementary file 4 — Supplementary Information 4. [file 41598_2022_5279_MOESM4_ESM.docx]

| **Supplementary Table 2.** Postoperative complications between PPCs (−) and (+) groups. | | | |
| --- | --- | --- | --- |
| Parameter | PPCs (−)  (n = 1,366) | PPCs (+)  (n = 178) | *P* value |
| Pulmonary complications |  |  |  |
| ARDS | 0 | 96 (54) |  |
| Pneumonia | 0 | 100 (56) |  |
| Atelectasis | 0 | 25 (14) |  |
| Arrhythmia | 262 (19) | 67 (37) | < 0.001 |
| Atrial fibrillation | 211 (15) | 55 (31) | < 0.001 |
| Others | 51 (4) | 12 (7) | 0.025 |
| Myocardial infarction | 3 (0) | 0 (0) | 0.529 |
| Empyema | 9 (1) | 12 (7) | < 0.001 |
| Wound infection | 20 (2) | 21 (12) | < 0.001 |
| Wound dehiscence | 5 (0) | 1 (1) | 0.701 |
| Bronchopleural fistula | 5 (0) | 11 (6) | < 0.001 |
| Prolonged air leak | 110 (8) | 11 (6) | 0.331 |
| Prolonged effusion | 19 (1) | 11 (6) | < 0.001 |
| Chylothorax | 24 (2) | 2 (1) | 0.489 |
| Vocal cord palsy | 53 (4) | 5 (3) | 0.415 |
| Acute renal injury* | 14 (1) | 26 (15) | < 0.001 |
| Cerebral infarction | 5 (0) | 2 (1) | 0.162 |
| PTE | 1 (0) | 2 (1) | 0.003 |
| Hospital stay | 8 (7-11) | 16 (12-34) | < 0.001 |
| ICU stay | 1 (1-2) | 5 (1-19) | < 0.001 |
| In-hospital mortality | 9 (0.7) | 30 (17) | < 0.001 |

Values are n (%) or median (interquartile range). *Acute renal injury was defined as Acute Kidney Injury Network classification 2 and 3.

PPCs, postoperative pulmonary complications; ARDS, acute respiratory distress syndrome; PTE, pulmonary thromboembolism; ICU, intensive care unit.
