# Supplementary material for: The impact of pulmonary function tests on early postoperative complications in open lung resection surgery: an observational cohort study
Source: Sci Rep. 2022 Jan 24;12:1277. doi: 10.1038/s41598-022-05279-8 (PMC8786949; doi:10.1038/s41598-022-05279-8)
Supplement: Supplementary file 5 — Supplementary Information 5. [file 41598_2022_5279_MOESM5_ESM.docx]

| **Supplementary Table 3.** Uni- and multivariable analysis for the risk factors for PPCs using the previous cutoffs. | | | | | | | | | | | | |
| --- | --- | --- | --- | --- | --- | --- | --- | --- | --- | --- | --- | --- |
| Variables | Univariable | | | Multivariable | | | | | | | | |
|  |  |  |  | Model 1 | | | Model 2 | | | Model 3 | | |
|  | OR | 95% CI | *P* value | OR | 95% CI | *P* value | OR | 95% CI | *P* value | OR | 95% CI | *P* value |
| ppoFEV1 or ppoDLCO < 40% (n=238) | 2.8 | 2.0 to 4.0 | < 0.001 |  |  |  | 2.060 | 1.374 to 3.088 | <0.001 |  |  |  |
| ppoFEV1 and ppoDLCO < 40% (n=74) | 2.6 | 1.5 to 4.6 | 0.002 |  |  |  |  |  |  | 1.878 | 0.987 to 3.575 | 0.055 |
| Age ≥ 66 years | 2.2 | 1.6 to 3.1 | < 0.001 | 1.784 | 1.246 to 2.554 | 0.002 | 1.861 | 1.295 to 2.672 | <0.001 | 1.838 | 1.281 to 2.638 | 0.001 |
| Male | 5.9 | 2.7 to 12.6 | < 0.001 | 3.677 | 1.669 to 8.102 | 0.001 | 3.372 | 1.525 to 7.452 | 0.003 | 3.597 | 1.632 to 7.932 | 0.002 |
| Current smoker | 1.7 | 1.2 to 2.5 | 0.003 | 1.534 | 1.049 to 2.242 | 0.027 | 1.592 | 1.085 to 2.334 | 0.017 | 1.547 | 1.058 to 2.264 | 0.025 |
| Transfusion | 3.7 | 2.7 to 5.1 | < 0.001 | 2.447 | 1.688 to 3.548 | <0.001 | 2.324 | 1.598 to 3.382 | <0.001 | 2.387 | 1.643 to 3.467 | <0.001 |
| Use of inotropes | 3.2 | 2.3 to 4.4 | < 0.001 | 2.051 | 1.410 to 2.985 | <0.001 | 1.902 | 1.301 to 2.781 | 0.001 | 1.976 | 1.353 to 2.885 | <0.001 |
| ASA physical status ≥ 3 | 2.1 | 1.3 to 3.2 | 0.001 |  |  |  |  |  |  |  |  |  |
| Heavy drinking | 1.4 | 0.9 to 2.2 | 0.147 |  |  |  |  |  |  |  |  |  |
| Body mass index, per kg/m^2^ | 0.9 | 0.9 to 1.0 | 0.035 |  |  |  |  |  |  |  |  |  |
| Diabetes mellitus | 1.1 | 0.7 to 1.6 | 0.779 |  |  |  |  |  |  |  |  |  |
| Hypertension | 1.0 | 0.7 to 1.4 | 0.902 |  |  |  |  |  |  |  |  |  |
| Previous lung operation | 1.9 | 0.8 to 4.6 | 0.175 |  |  |  |  |  |  |  |  |  |
| Pulmonary disease | 2.3 | 1.5 to 3.5 | < 0.001 |  |  |  |  |  |  |  |  |  |
| Cerebrovascular disease | 2.2 | 1.2 to 4.3 | 0.018 |  |  |  |  |  |  |  |  |  |
| Heart disease | 0.8 | 0.4 to 1.7 | 0.580 |  |  |  |  |  |  |  |  |  |
| Pulmonary tuberculosis | 1.3 | 0.8 to 1.9 | 0.321 |  |  |  |  |  |  |  |  |  |
| Neoadjuvant CCRT | 1.3 | 0.9 to 1.9 | 0.118 |  |  |  |  |  |  |  |  |  |
| Cell type |  |  |  |  |  |  |  |  |  |  |  |  |
| Squamous cell carcinoma | reference | | |  |  |  |  |  |  |  |  |  |
| Adenocarcinoma | 0.6 | 0.4 to 0.8 | 0.001 |  |  |  |  |  |  |  |  |  |
| Etc. | 0.7 | 0.4 to 1.4 | 0.338 |  |  |  |  |  |  |  |  |  |
| TNM stage 3& 4 | 1.1 | 0.8 to 1.6 | 0.437 |  |  |  |  |  |  |  |  |  |
| Operation |  |  |  |  |  |  |  |  |  |  |  |  |
| Lobectomy | reference | | |  |  |  |  |  |  |  |  |  |
| Sleeve lobectomy | 0.8 | 0.5 to 1.4 | 0.502 |  |  |  |  |  |  |  |  |  |
| Left pneumonectomy | 1.3 | 0.7 to 2.2 | 0.426 |  |  |  |  |  |  |  |  |  |
| Right pneumonectomy | 2.3 | 1.2 to 4.3 | 0.013 |  |  |  |  |  |  |  |  |  |
| Postoperative analgesia |  |  |  |  |  |  |  |  |  |  |  |  |
| Thoracic epidural analgesia | reference | | |  |  |  |  |  |  |  |  |  |
| IV-PCA | 0.9 | 0.6 to 1.3 | 0.491 |  |  |  |  |  |  |  |  |  |
| Paravertebral block | 1.3 | 0.9 to 1.9 | 0.180 |  |  |  |  |  |  |  |  |  |
| Use of vasopressors | 1.8 | 1.3 to 2.5 | < 0.001 |  |  |  |  |  |  |  |  |  |
| Use of hydroxyethyl starch | 2.3 | 1.7 to 3.2 | < 0.001 |  |  |  |  |  |  |  |  |  |
| Operation duration ≥ 3 h | 1.6 | 1.1 to 2.4 | 0.020 |  |  |  |  |  |  |  |  |  |
| AUC_ROC_ |  |  |  | 0.737 | 0.695 to 0.778 | < 0.001 | 0.751 | 0.712 to 0.791 | < 0.001 | 0.738 | 0.696 to 0.779 | < 0.001 |

PPCs, postoperative pulmonary complications; ppoFEV1, predicted postoperative forced expiratory volume in 1 second; ppoDLCO, predicted postoperative diffusing capacity for carbon monoxide; ASA, American Society of Anesthesiologist; CCRT, concurrent chemoradiotherapy; TNM, tumor node metastasis; IV-PCA, intravenous patient controlled analgesia; AUC_ROC_, area under the curve of the receiver operating characteristic curve.
