# Supplementary material for: The impact of pulmonary function tests on early postoperative complications in open lung resection surgery: an observational cohort study
Source: Sci Rep. 2022 Jan 24;12:1277. doi: 10.1038/s41598-022-05279-8 (PMC8786949; doi:10.1038/s41598-022-05279-8)
Supplement: Supplementary file 6 — Supplementary Information 6. [file 41598_2022_5279_MOESM6_ESM.docx]

| **Supplementary Table 4.** Propensity score matching between the Above and Below groups. | | | | | | | | | | | | | | | | | |
| --- | --- | --- | --- | --- | --- | --- | --- | --- | --- | --- | --- | --- | --- | --- | --- | --- | --- |
| Variables | Data before matching | | | | | 1:1 matched data | | | | | | | | | | | |
|  |  |  |  |  |  | PPCs  Matching 1 (caliper = 0.3) | | | | In-hospital mortality  Matching 2 (caliper = 0.2) | | | | Long-term survival  Matching 3 (caliper = 0.4) | | | |
|  | Level | Group | | P value | SMD | Group | | P value | SMD | Group | | P value | SMD | Group | | P value | SMD |
|  |  | Above | Below |  |  | Above | Below |  |  | Above | Below |  |  | Above | Below |  |  |
|  |  | 1173 | 248 |  |  | 160 | 160 |  |  | 241 | 241 |  |  | 178 | 178 |  |  |
| Age ≥ 66 years, n (%) | 0 | 619 (52.8) | 146 (58.9) | 0.093 | 0.123 | 91 (56.9) | 86 (53.8) | 0.653 | 0.063 | 141 (58.5) | 139 (57.7) | 0.926 | 0.017 | 98 (55.1) | 98 (55.1) | 1 | <0.001 |
|  | 1 | 554 (47.2) | 102 (41.1) |  |  | 69 (43.1) | 74 (46.2) |  |  | 100 (41.5) | 102 (42.3) |  |  | 80 (44.9) | 80 (44.9) |  |  |
| Male, n (%) | 0 | 228 (19.4) | 18 (7.3) | <0.001 | 0.364 | 10 (6.2) | 14 (8.8) | 0.524 | 0.095 | 17 ( 7.1) | 18 ( 7.5) | 1 | 0.016 | 17 ( 9.6) | 14 (7.9) | 0.707 | 0.06 |
|  | 1 | 945 (80.6) | 230 (92.7) |  |  | 150 (93.8) | 146 (91.2) |  |  | 224 (92.9) | 223 (92.5) |  |  | 161 (90.4) | 164 (92.1) |  |  |
| BMI, mean (SD) |  | 23.54 (3.08) | 23.14 (3.62) | 0.110 | 0.118 | 23.50 (2.96) | 23.48 (3.80) | 0.966 | 0.005 | 23.34 (3.16) | 23.19 (3.64) | 0.636 | 0.043 | 23.33 (2.75) | 23.21 (3.74) | 0.734 | 0.036 |
| ASA physical status ≥ 3, n (%) | 0 | 1085 (92.5) | 204 (82.3) | <0.001 | 0.312 | 126 (78.8) | 132 (82.5) | 0.479 | 0.095 | 203 (84.2) | 204 (84.6) | 1 | 0.011 | 147 (82.6) | 147 (82.6) | 1 | <0.001 |
|  | 1 | 88 (7.5) | 44 (17.7) |  |  | 34 (21.2) | 28 (17.5) |  |  | 38 (15.8) | 37 (15.4) |  |  | 31 (17.4) | 31 (17.4) |  |  |
| Current smoker, n (%) | 0 | 761 (64.9) | 164 (66.1) | 0.931 | 0.027 | 117 (73.1) | 120 (75.0) | 0.799 | 0.043 | 136 (56.4) | 159 (66.0) | 0.097 | 0.198 | 111 (62.4) | 113 (63.5) | 0.975 | 0.024 |
|  | 1 | 244 (20.8) | 50 (20.2) |  |  | 43 (26.9) | 40 (25.0) |  |  | 63 (26.1) | 48 (19.9) |  |  | 43 (24.2) | 42 (23.6) |  |  |
|  | NA | 168 (14.3) | 34 (13.7) |  |  |  |  |  |  | 42 (17.4) | 34 (14.1) |  |  | 24 (13.5) | 23 (12.9) |  |  |
| Heavy drinking, n (%) | 0 | 1036 ( 88.3) | 214 ( 86.3) | 0.432 | 0.061 | 142 (88.8) | 139 (86.9) | 0.733 | 0.057 | 206 (85.5) | 209 (86.7) | 0.792 | 0.036 | 154 (86.5) | 154 (86.5) | 1 | <0.001 |
|  | 1 | 137 (11.7) | 34 (13.7) |  |  | 18 (11.2) | 21 (13.1) |  |  | 35 (14.5) | 32 (13.3) |  |  | 24 (13.5) | 24 (13.5) |  |  |
| Cell types, n (%) | 1 | 551 (47.0) | 170 (68.5) | <0.001 | 0.461 | 107 (66.9) | 106 (66.2) | 0.877 | 0.057 | 166 (68.9) | 163 (67.6) | 0.861 | 0.05 | 112 (62.9) | 115 (64.6) | 0.68 | 0.093 |
|  | 2 | 521 (44.4) | 60 (24.2) |  |  | 38 (23.8) | 41 (25.6) |  |  | 60 (24.9) | 60 (24.9) |  |  | 56 (31.5) | 50 (28.1) |  |  |
|  | 3 | 101 (8.6) | 18 (7.3) |  |  | 15 ( 9.4) | 13 ( 8.1) |  |  | 15 ( 6.2) | 18 ( 7.5) |  |  | 10 (5.6) | 13 (7.3) |  |  |
| TNM stage 3 & 4, n (%) | 0 | 803 (68.5) | 142 (57.3) | 0.001 | 0.279 | 118 (73.8) | 96 (60.0) | 0.013 | 0.295 | 142 (58.9) | 142 (58.9) | 1 | <0.001 | 114 (64.0) | 113 (63.5) | 1 | 0.012 |
|  | 1 | 360 (30.7) | 106 (42.7) |  |  | 42 (26.2) | 64 (40.0) |  |  | 99 (41.1) | 99 (41.1) |  |  | 64 (36.0) | 65 (36.5) |  |  |
|  | NA | 10 (0.9) | 0 (0.0) |  |  |  |  |  |  |  |  |  |  |  |  |  |  |
| Neoadjuvant CCRT, n (%) | 0 | 936 (79.8) | 186 (75.0) | 0.11 | 0.115 | 126 (78.8) | 116 (72.5) | 0.241 | 0.146 | 188 (78.0) | 181 (75.1) | 0.519 | 0.069 | 133 (74.7) | 138 (77.5) | 0.619 | 0.066 |
|  | 1 | 237 ( 20.2) | 62 ( 25.0) |  |  | 34 (21.2) | 44 (27.5) |  |  | 53 (22.0) | 60 (24.9) |  |  | 45 (25.3) | 40 (22.5) |  |  |
| Hypertension, n (%) | 0 | 725 (61.8) | 157 (63.3) | 0.711 | 0.031 | 112 (70.0) | 94 (58.8) | 0.047 | 0.237 | 148 (61.4) | 154 (63.9) | 0.638 | 0.051 | 106 (59.6) | 110 (61.8) | 0.745 | 0.046 |
|  | 1 | 448 (38.2) | 91 (36.7) |  |  | 48 (30.0) | 66 (41.2) |  |  | 93 (38.6) | 87 (36.1) |  |  | 72 (40.4) | 68 (38.2) |  |  |
| Diabetes mellitus, n (%) | 0 | 980 (83.5) | 205 (82.7) | 0.805 | 0.024 | 128 (80.0) | 127 (79.4) | 1 | 0.016 | 190 (78.8) | 199 (82.6) | 0.356 | 0.095 | 144 (80.9) | 147 (82.6) | 0.784 | 0.044 |
|  | 1 | 193 (16.5) | 43 (17.3) |  |  | 32 (20.0) | 33 (20.6) |  |  | 51 (21.2) | 42 (17.4) |  |  | 34 (19.1) | 31 (17.4) |  |  |
| Previous lung operation, n (%) | 0 | 1154 ( 98.4) | 239 ( 96.4) | 0.069 | 0.126 | 157 (98.1) | 156 (97.5) | 1 | 0.043 | 234 (97.1) | 232 (96.3) | 0.799 | 0.046 | 175 (98.3) | 173 (97.2) | 0.723 | 0.076 |
|  | 1 | 19 (1.6) | 9 (3.6) |  |  | 3 ( 1.9) | 4 ( 2.5) |  |  | 7 (2.9) | 9 (3.7) |  |  | 3 (1.7) | 5 (2.8) |  |  |
| Previous lung disease, n (%) | 0 | 1059 (90.3) | 197 (79.4) | <0.001 | 0.306 | 127 (79.4) | 127 (79.4) | 1 | <0.001 | 216 (89.6) | 192 (79.7) | 0.004 | 0.279 | 156 (87.6) | 140 (78.7) | 0.034 | 0.242 |
|  | 1 | 114 (9.7) | 51 (20.6) |  |  | 33 (20.6) | 33 (20.6) |  |  | 25 (10.4) | 49 (20.3) |  |  | 22 (12.4) | 38 (21.3) |  |  |
| Cardiac disease, n (%) | 0 | 1113 (94.9) | 231 (93.1) | 0.345 | 0.073 | 142 (88.8) | 150 (93.8) | 0.166 | 0.178 | 224 (92.9) | 227 (94.2) | 0.71 | 0.051 | 160 (89.9) | 168 (94.4) | 0.168 | 0.168 |
|  | 1 | 60 (5.1) | 17 (6.9) |  |  | 18 (11.2) | 10 ( 6.2) |  |  | 17 (7.1) | 14 (5.8) |  |  | 18 (10.1) | 10 (5.6) |  |  |
| Cerebrovascular disease, n (%) | 0 | 1133 (96.6) | 238 (96.0) | 0.769 | 0.033 | 155 (96.9) | 152 (95.0) | 0.571 | 0.095 | 234 (97.1) | 231 (95.9) | 0.621 | 0.068 | 170 (95.5) | 168 (94.4) | 0.809 | 0.051 |
|  | 1 | 40 (3.4) | 10 (4.0) |  |  | 5 (3.1) | 8 (5.0) |  |  | 7 (2.9) | 10 (4.1) |  |  | 8 (4.5) | 10 (5.6) |  |  |
| Pulmonary tuberculosis, n (%) | 0 | 1024 (87.3) | 214 (86.3) | 0.744 | 0.03 | 134 (83.8) | 138 (86.2) | 0.639 | 0.07 | 213 (88.4) | 209 (86.7) | 0.679 | 0.05 | 155 (87.1) | 154 (86.5) | 1 | 0.017 |
|  | 1 | 149 (12.7) | 34 (13.7) |  |  | 26 (16.2) | 22 (13.8) |  |  | 28 (11.6) | 32 (13.3) |  |  | 23 (12.9) | 24 (13.5) |  |  |
| Type of surgery, n (%) | 1 | 971 (82.8) | 115 (46.4) | <0.001 | 1.164 | 101 (63.1) | 98 (61.3) | 0.89 | 0.089 | 187 (77.6) | 113 (46.9) | <0.001 | 1.119 | 119 (66.9) | 115 (64.6) | 0.833 | 0.099 |
|  | 2 | 151 (12.9) | 13 (5.2) |  |  | 13 (8.1) | 12 (7.5) |  |  | 40 (16.6) | 12 (5.0) |  |  | 9 (5.1) | 13 (7.3) |  |  |
|  | 3 | 43 (3.7) | 69 (27.8) |  |  | 39 (24.4) | 40 (25.0) |  |  | 12 (5.0) | 68 (28.2) |  |  | 42 (23.6) | 41 (23.0) |  |  |
|  | 4 | 8 (0.7) | 51 (20.6) |  |  | 7 (4.4) | 10 ( 6.2) |  |  | 2 (0.8) | 48 (19.9) |  |  | 8 (4.5) | 9 (5.1) |  |  |
| Postoperative analgesia, n (%) | 1 | 365 (31.1) | 91 (36.7) | 0.025 | 0.195 | 50 (31.2) | 52 (32.5) | 0.307 | 0.172 | 87 (36.1) | 88 (36.5) | 0.867 | 0.049 | 71 (39.9) | 66 (37.1) | 0.666 | 0.096 |
|  | 2 | 411 (35.0) | 65 (26.2) |  |  | 54 (33.8) | 42 (26.2) |  |  | 69 (28.6) | 64 (26.6) |  |  | 52 (29.2) | 49 (27.5) |  |  |
|  | 3 | 397 (33.8) | 92 (37.1) |  |  | 56 (35.0) | 66 (41.2) |  |  | 85 (35.3) | 89 (36.9) |  |  | 55 (30.9) | 63 (35.4) |  |  |

One-to-one matching was performed using the nearest-neighbor method. Variables were well balanced after matching. 0, No and 1, Yes in binary variables; NA, Non applicable; Cell types, 1 Squamous cell carcinoma, 2 Adenocarcinoma, and 3 etc.; Type of surgery, 1 Lobectomy, 2 Sleeve lobectomy, 3 Left pneumonectomy, and 4 Right pneumonectomy; Postoperative analgesia, 1 Thoracic epidural analgesia, 2 Intravenous patient controlled analgesia, and 3 Paravertebral block.

PPCs, postoperative pulmonary complications; SMD, standard mean difference; BMI, body mass index; ASA, American Society of Anesthesiologist; TNM, tumor node metastasis; CCRT, concurrent chemoradiotherapy.
