# Supplementary material for: The impact of pulmonary function tests on early postoperative complications in open lung resection surgery: an observational cohort study
Source: Sci Rep. 2022 Jan 24;12:1277. doi: 10.1038/s41598-022-05279-8 (PMC8786949; doi:10.1038/s41598-022-05279-8)
Supplement: Supplementary file 7 — Supplementary Information 7. [file 41598_2022_5279_MOESM7_ESM.docx]

**The impact of pulmonary function tests on early postoperative complications in open lung resection surgery: An observational cohort study**

**Abbreviated Title:** Pulmonary function tests in thoracotomy

Ji Won Choi^1,†^, Heejoon Jeong^1,†^, Hyun Joo Ahn^1,*^, Mikyung Yang^1^, Jie Ae Kim^1^, Duk Kyung Kim^1^, Sang Hyun Lee^1^, Keoungah Kim^2^, Jisun Choi^1^

^1^Department of Anesthesiology and Pain Medicine, Samsung Medical Center, Sungkyunkwan University School of Medicine, Seoul, South Korea

^2^Department of Anesthesiology, School of Dentistry, Dankook University, Cheonan, South Korea

***Corresponding author**: Hyun Joo Ahn

Address: 81 Irwon-ro, Gangnam-gu, Seoul 06351, South Korea

Tel: +82-2-3410-0784, Fax: +82-2-3410-6626, E-mail: hyunjooahn@skku.edu

†Ji Won Choi and Heejoon Jeong equally contributed as co-first authors.

**Supplementary legends**

**Supplementary Table 1.** Baseline patient and operative characteristics, compared between the PPC (−) and PPC (+) groups.

PPCs, postoperative pulmonary complications.

**Supplementary Table 2.** Postoperative complications between PPCs (−) and (+) groups.

PPCs, postoperative pulmonary complications.

**Supplementary Table 3.** Uni- and multivariable analysis for the risk factors for PPCs using the previous cutoffs. AUC_ROC_s of three different models were presented; model 1 (only co-variates), model 2 (model 1 + ppoFEV1 or ppoDLco < 40%), and model 3 (model 1 + ppoFEV1 and ppoDLco < 40%)

PPCs, postoperative pulmonary complications.

**Supplementary Table 4.** Propensity score matching between Above and Below groups.

**Supplementary Figure 1.** Propensity matching plots for PPCs in Above and Below groups.

PPCs, postoperative pulmonary complications.

**Supplementary Figure 2** Multivariable AUC_ROC_s of the three models. Model 1, covariates (age ≥ 66 years, male sex, current smoker, intraoperative transfusion and use of inotropes); Model 2, model 1 + ppoFEV1 or ppoDLco < 40%; Model 3, model 1 + ppoFEV1 and ppoDLco < 40%.

AUC_ROC_: Area under the receiver operating characteristic curve. CI: confidence interval. ppoFEV_1_: postoperative expiratory volume in 1 second. ppoDL_CO_: diffusing capacity for carbon monoxide.
